# Cardiopulmonary Consequences of Scoliosis and the Clinical Implications of VO_2_max: A Systematic Review

**DOI:** 10.1016/j.jposna.2026.100369

**Published:** 2026-04-02

**Authors:** Suhani Sharma, Arun Ramnarine, Karim Fouad, Samuel Kirby, Shreya Sankar, Michael O'Connor, George-Paul O'Byrne, Oliver Boughton, Patrick Kiely, Tony Rafferty

**Affiliations:** 1 School of Medicine, Royal College of Surgeons in Ireland, Dublin, Ireland; 2 Department of Surgery, University Hospital Limerick, Limerick, Ireland; 3 Scoliosis Academy, CHI Crumlin, Dublin, Ireland

**Keywords:** Adolescent idiopathic scoliosis, Cardiopulmonary exercise testing, VO_2_max

## Abstract

**Introduction:**

Multiple parameters, including VO_2_max, ventilatory efficiency, lung volumes, and exercise tolerance, have been examined, yet their progression with pediatric scoliosis remains unclear. Although pulmonary function tests are widely used, they may underestimate functional impairment, as spirometric measures can remain relatively preserved despite clinically meaningful reductions in exercise capacity. This review aims to define the extent of functional impairment, its association with curve severity, and the potential of VO_2_max as a marker of surgical recovery.

**Materials and methods:**

We systematically searched PubMed, Embase, and Cochrane for eligible studies reporting cardiopulmonary outcomes in pediatric scoliosis. Risk of bias was assessed using the Joanna Briggs Institute critical appraisal tools and the ROBINS-I risk-of-bias tool. Results were synthesized narratively following the Synthesis Without Meta-analysis guideline.

**Conclusions:**

From 455 records, 6 studies met the inclusion criteria. Adolescents with idiopathic scoliosis consistently demonstrated reduced aerobic capacity, with mean VO_2_max values ranging from 27.9 to 33.6 mL kg^−1^·min^−1^, approximately 20-25% below reference norms, indicating mild-to-moderate cardiopulmonary impairment. Functional tests such as the 6-min walk and E-fit correlated strongly with VO_2_max, validating their use as practical surrogates of aerobic performance. Greater curve severity and thoracic deformity were associated with progressive reductions in forced expiratory volume in one second, forced vital capacity, and VO_2_max, reflecting restrictive ventilatory physiology. Postoperative studies showed stable but not improved VO_2_ or pulmonary function, suggesting that surgery preserves rather than enhances respiratory capacity. Exercise-based rehabilitation improved posture and endurance, though effects on VO_2_max remain inconclusive.

A spectrum of cardiopulmonary parameters deteriorates with scoliosis progression. VO_2_ max may provide the most integrative marker of physiological burden, and prospective longitudinal data are required to establish its role in recovery after surgery.

**Key Concept:**

(1) Adolescents with scoliosis exhibit clinically meaningful reductions in cardiopulmonary reserve, which may not be apparent on resting spirometry but become evident during exercise and directly influence perioperative physiological resilience.
(2) Radiographic measures of deformity correction do not reliably reflect functional recovery, underscoring the limitations of relying on Cobb angle and pulmonary function tests alone when assessing surgical readiness and postoperative outcomes.
(3) Cardiopulmonary exercise testing (CPET) provides an objective, integrative assessment of surgical fitness, allowing differentiation between ventilatory, cardiovascular, and peripheral contributors to exercise limitation, with VO_2_max serving as a robust summary metric.
(4) Corrective scoliosis surgery appears to preserve rather than restore cardiopulmonary function, suggesting that preoperative physiological status may be a key determinant of postoperative recovery trajectory and length of convalescence.
(5) Incorporation of VO_2_max into preoperative assessment and Enhanced Recovery After Surgery pathways may enhance risk stratification, inform prehabilitation strategies, and guide postoperative rehabilitation, enabling more individualized perioperative care for children and adolescents undergoing scoliosis surgery.

## Introduction

Scoliosis is a complex three-dimensional deformity of the spine and rib cage that alters thoracic geometry, reduces chest wall compliance, and compromises lung expansion. These anatomical changes increase the work of breathing and predispose patients to restrictive ventilatory patterns, impaired gas exchange, and reduced functional reserve [1]. Pulmonary function tests (PFTs), particularly forced vital capacity (FVC), forced expiratory volume in one second (FEV_1_), and total lung capacity (TLC), are traditionally employed to assess these deficits. However, although reductions in lung volumes correlate with increasing curve severity [2], spirometric measures often remain deceptively preserved in patients who nonetheless experience significant functional limitation [3].

Recognizing this limitation, research has turned to functional exercise tests to better capture real-world impairment. The 6-min walk test (6MWT) and incremental shuttle walk test are practical, reproducible measures that reflect submaximal aerobic capacity [[4], [5], [6]]. Building on these observations, cardiopulmonary exercise testing (CPET) has emerged as the gold standard for assessing integrated cardiovascular, respiratory, and muscular responses to exertion [7]. CPET provides insight not only into ventilatory limitation but also into oxygen delivery and utilization, with maximal oxygen uptake (VO_2_max or VO_2_peak) serving as a key indicator of aerobic capacity. In adolescent idiopathic scoliosis (AIS), VO_2_peak values are reduced by up to 20-30% relative to controls, with emerging evidence suggesting that impairment relates not solely to Cobb angle magnitude but to the three-dimensional nature of the deformity, including axial rotation and rib cage distortion [8]. Additional analyses have linked chest wall rotational deformity with further reductions in peak VO_2_, underscoring the anatomical–functional relationship [9]. Beyond pulmonary limitations, scoliosis may also impose direct cardiovascular consequences. Echocardiographic studies in severe thoracic scoliosis have demonstrated reduced left ventricular dimensions, diastolic diameters, and cardiac index, with negative correlations with Cobb angle severity [10]. These findings suggest that scoliosis is not solely a restrictive pulmonary disorder but may also affect cardiac structure and function, thereby compounding deficits in exercise capacity and recovery potential.

Taken together, the current evidence highlights a discordance between traditional resting assessments and the lived functional impairment of patients with scoliosis. PFTs, while widely available, incompletely reflect physiological burden imposed by thoracic deformity during physical exertion, when cardiopulmonary limitations are most pronounced [7,11]. CPET, the most comprehensive modality, is reported in only a small fraction of studies, and its prognostic implications remain largely unexplored. In particular, the role of VO_2_max as a predictor of perioperative risk and recovery in scoliosis surgery is understudied, representing a major gap in both clinical practice and research.

Accordingly, this systematic review synthesizes available evidence on cardiopulmonary function in pediatric scoliosis, with emphasis on CPET-derived measures such as VO_2_max. We hypothesized that adolescents with scoliosis demonstrate reduced aerobic capacity relative to healthy peers and that this reduction correlates with deformity severity. In accordance with our published protocol [12], we further aimed to evaluate whether VO_2_max functions as an indicator of surgical outcomes and a postoperative cardiopulmonary metric in pediatric scoliosis. Given the heterogeneity and limited scope of existing studies, we also sought to determine whether current evidence adequately supports these objectives or instead identifies important gaps requiring prospective investigation.

## Methods

### Search strategy and selection process

A comprehensive systematic literature search was conducted across PubMed, Embase, and Cochrane from database inception up to June 23, 2025 to identify eligible studies in accordance with the inclusion and exclusion criteria (Table 1). The search strategy combined MeSH terms and free-text keywords, which can be found in Appendix A. Studies published in languages other than English were excluded in accordance with the predefined protocol [12] due to resource constraints and the need to ensure accurate interpretation of technical cardiopulmonary methodology.

**Table 1:** Eligibility criteria.

PICO component	Inclusion criteria	Exclusion criteria
Population (P)	- Patients aged 0-18 y diagnosed with idiopathic, congenital, or neuromuscular scoliosis. - Individuals undergoing or considered for surgical correction of scoliosis.	- Adults (>18 y) - Studies involving nonscoliotic populations. - Animal or cadaveric studies.
Intervention/exposure (I)	- Presence of scoliosis with potential cardiopulmonary compromise due to thoracic deformity. - Studies assessing preoperative or postoperative cardiopulmonary function.	Interventions unrelated to scoliosis (eg respiratory rehabilitation in nonscoliotic patients).
Comparator (C)	- Healthy age-matched controls. - Comparisons between severities of scoliosis, curve types, or pre- vs post-operative states.	Studies without a comparator group or relevant subgroup analysis (unless longitudinal).
Outcomes (O)	- Quantitative cardiopulmonary outcomes, including: ○ VO_2_max/VO_2_peak (CPET) ○ Pulmonary function tests (FVC, FEV_1_, TLC) ○ 6-min walk test (6MWT) or equivalent functional tests. ○ Clinical associations with recovery, complications, hospital stay, rehabilitation, ambulation, or quality of life (eg SRS-22, ERAS outcomes).	- Studies focusing exclusively on radiological, surgical, or biomechanical parameters without cardiopulmonary measures. - Qualitative or nonquantitative outcomes.
Study design (S)	Clinical trials, prospective or retrospective cohort studies, cross-sectional studies, and systematic reviews/meta-analyses providing extractable primary data.	- Case reports, case series, editorials, letters, and conference abstracts. - Non-English studies. - Unavailable full-text publications.

*CPET, cardiopulmonary exercise testing; FVC, forced vital capacity; FEV***_*1*_***, forced expiratory volume in one second; TLC, total lung capacity*.

### Eligibility criteria

The inclusion and exclusion criteria are detailed in Table 1**.**

### Data extraction

For each included study, the following information was extracted: study characteristics (author, year of publication, country, study type, and study title), population details (sample size, mean age, prevalence of asthma, and body mass index [BMI]), and outcome measures. Extracted outcomes comprised preoperative and mean VO_2_max values (mL/kg/min and % predicted), pulmonary function indices (FEV_1_, FVC, TLC), spinal deformity parameters (coronal and thoracic curvature, thoracic kyphosis, and percentage curve correction), and postoperative recovery metrics (eg length of stay).

### Synthesis methods

Grouping decisions were based on population and outcome characteristics to enable a transparent, structured narrative synthesis consistent with the Synthesis Without Meta-analysis (SWiM) guideline [13], as outlined in our protocol [12].

### Study of risk of bias assessment

For observational cohort studies, the ROBINS-I tool [14] was applied to evaluate the risk of bias for each clinical outcome relevant to the review's objectives. For cross-sectional, case-control studies, as well as systematic reviews, the Joanna Briggs Institute (JBI) Critical Appraisal Checklist [15] was used to ensure consistent and rigorous appraisal across study types.

## Results

### Study selection

**Figure 1:**
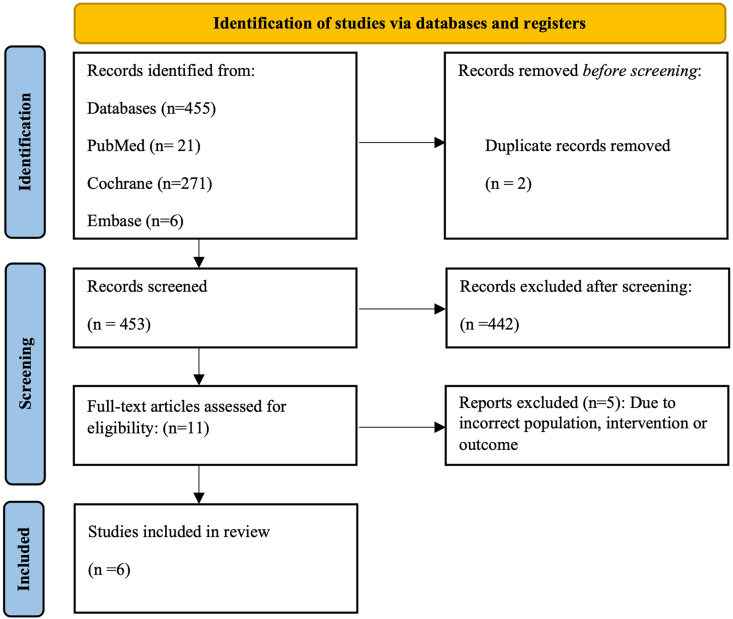
PRISMA literature search flowchart. *PRISMA, Preferred Reporting Items for Systematic reviews and Meta-Analyses.*

A total of 455 records were identified across 3 electronic databases (Fig. 1). After 2 duplicates were removed, 453 studies underwent title and abstract screening. Of these, 11 full-text articles were assessed for eligibility. Ultimately, 6 studies met the inclusion criteria and were included in the narrative synthesis.

### Study characteristics

Studies included were either prospective or observational cohort studies (Shen et al., [16] Lin et al., [17] Guo et al. [18]), one cross-sectional study (Siwiec et al. [8]), one case–control study (Lau et al. [19]), and one retrospective review (Farrell et al. [20]). Sample sizes ranged from 22 to 170 participants, with mean ages between 12 and 20 years. Females represented approximately 65-80% of each cohort. Most studies were conducted in China and primarily investigated adolescents with idiopathic or congenital scoliosis using CPET, spirometry, or functional performance measures. Detailed study characteristics are summarized in Appendix B.

### VO_2_max and aerobic performance in scoliosis

Across all CPET-based studies, participants with AIS demonstrated reduced aerobic capacity compared with age-matched controls or published reference ranges. Shen et al. (2016) [16] reported a mean VO_2_max of *27.9 ± 5.*2 mL kg^−1^·min^−1^ in female AIS patients, whereas Lin et al. (2016) [17] found *33.5 ± 9.*6 mL kg^−1^·min^−1^ in males–both values falling substantially below those expected for healthy adolescents of comparable age and sex. Siwiec et al. (2024) [8] reported a VO_2_peak of 33.6 ± 7.5 mL kg^−1^·min^−1^ in AIS participants vs 43.0 ± 8.3 mL kg^−1^·min^−1^ in controls, representing an approximate 20-25% reduction in aerobic capacity [8]. Lau et al. (2023) [19] similarly observed elevated oxygen consumption and delayed attainment of VO_2_peak during an electronic-fit (E-Fit) protocol among AIS participants compared with controls [19], indicating impaired oxygen utilization efficiency.

When expressed as a proportion of predicted values, VO_2_max typically ranged between 78-80% of predicted, while FEV_1_ and FVC values were approximately 85-90% of predicted. Together, these findings indicate that adolescents with AIS demonstrate mild-to-moderate reductions in aerobic capacity and pulmonary reserve relative to peers without spinal deformity.

### Functional surrogates of VO_2_: 6-min walk and E-Fit performance

Guo et al. (2024) [18] examined the 6-min walk test (6MWT) as a practical alternative to CPET and identified a strong positive correlation between walking distance and measured VO_2_max, validating the 6MWT as a surrogate measure of aerobic performance in children with scoliosis. Similarly, Lau et al. (2023) [19] demonstrated that adolescents with mild-to-moderate AIS exhibited higher oxygen consumption and metabolic cost during E-Fit sessions despite equivalent external workloads, indicating increased physiological effort to sustain comparable activity levels. Both studies suggest that functional endurance tests reliably reflect underlying aerobic limitation in scoliosis and may serve as accessible tools for clinical or postoperative monitoring.

### Structural deformity and cardiorespiratory performance

Several observational cohorts explored the relationship between spinal curvature and cardiorespiratory parameters. Findings consistently showed that greater thoracic deformity correlates with reduced pulmonary function and aerobic capacity. Shen et al. (2016) [16] reported a negative correlation between thoracic kyphosis and CPET results (*r* = −0.28, *P* = .12), while Lin et al. (2016) [17] and Siwiec et al. (2024) [8] each noted progressive declines in FEV_1_ and FVC with increasing Cobb angle severity. These results support the mechanistic hypothesis that thoracic cage restriction from spinal curvature contributes to diminished ventilatory efficiency and oxygen uptake, with functional impairment becoming more pronounced as deformity magnitude increases.

Although absolute VO_2_ values varied across cohorts–likely reflecting differences in participant demographics and exercise protocols–studies converged on the conclusion that restrictive ventilatory physiology underlies the reduced VO_2_max observed in scoliosis populations.

### Postoperative and longitudinal changes in VO_2_ and pulmonary function

Siwiec et al. (2024) [8] demonstrated that in adolescents with idiopathic scoliosis, posterior corrective surgery does not significantly improve long-term respiratory or cardiopulmonary function despite follow-up periods of at least two years [8]. While absolute lung volumes (FVC and FEV_1_) may rise after surgery, these increases reflect normal growth rather than a true functional gain (percent-predicted values remain unchanged). Similarly, studies assessing oxygen uptake (VO_2_) and exercise capacity show little to no postoperative improvement, indicating that dynamic cardiopulmonary performance often remains below that of healthy peers. Over time, pulmonary function tends to remain stable rather than deteriorate, suggesting that surgery may help preserve but not enhance respiratory capacity or VO_2_ efficiency.

### Overall synthesis and certainty of evidence

Across all included studies, adolescents with idiopathic scoliosis consistently demonstrated reduced VO_2_max or VO_2_peak values and altered exercise efficiency compared with healthy controls (Table 2). Functional tests such as the 6MWT correlated positively with directly measured VO_2_, providing pragmatic assessment options when formal CPET is unavailable. The consistency in direction of effect across heterogeneous study designs supports an association between scoliosis and reduced aerobic capacity.

**Table 2:** Summary of included studies and key findings.

Study (author, year)	Population and design	Outcome measure(s)	Comparator(s)	Main findings/summary
Shen et al., 2016	Adolescents with idiopathic scoliosis (33 females, 7 males); prospective cohort using CPET.	**VO_2_max (mL·kg^−1^·min^−1^), PFTs (FEV_1_, FVC).**	Reference norms for healthy adolescents.	**Mean VO_2_max 27.9**±**nbsp;5.2 mL kg^−1^·min^−1^,** lower than predicted; larger curves associated with worse PFTs; regular exercise improved VO_2_peak.
Lin et al., 2019	Adolescents with congenital scoliosis (60 total: 19 males, 41 females); observational cohort.	**VO_2_max, FEV_1_, FVC,** TLC.	Internal group comparison by curve severity.	**VO_2_max inversely correlated with thoracic kyphosis**; severe deformity associated with restrictive pattern and reduced exercise capacity.
Guo et al., 2024	Children with idiopathic scoliosis (n = 65); prospective cohort using 6-minute walk test.	**6MWT distance, VO_2_max correlation.**	Internal correlation and normative data.	**Strong positive correlation between 6MWT and VO_2_max (r = 0.43, *P* < .001);** supports 6MWT as surrogate for aerobic capacity.
Siwiec et al., 2024	Adolescents with idiopathic scoliosis (n = 92; 63 F, 29 M); cross-sectional CPET comparison.	**VO_2_peak,** ventilatory efficiency.	Healthy age-matched controls.	**VO_2_peak 33.6**plusmn;**nbsp;7.5 vs 43.0**±**nbsp;8.3 mL kg^−1^**·**min^−1^;** AIS group showed **∼20-25% lower aerobic capacity** and shorter test duration.
Lau et al., 2023	Adolescents with mild-to-moderate AIS (n = 22 F); case–control using E-Fit functional testing.	**VO_2_peak,** oxygen consumption, metabolic cost.	Healthy controls.	AIS group had **higher oxygen consumption and metabolic cost** during E-Fit; reduced efficiency despite comparable workload.
Farrell et al., 2020	Adolescents with thoracic AIS (n = 170; mean age 15 y); retrospective review.	**PFTs (FEV_1_, FVC),** radiographic predictors.	Preoperative radiographic parameters.	Lower diaphragmatic domes and hypokyphosis correlated with reduced lung volumes; **convex hemi-thoracic width had stronger association with FEV_1_ and FVC than Cobb angle.**

*CPET, cardiopulmonary exercise testing; AIS, adolescents with idiopathic scoliosis; PFTs, pulmonary function tests; FEV***_*1*_***, forced expiratory volume in one second; FVC, forced vital capacity; TLC, total lung capacity*.

Using GRADE-informed principles, the overall certainty of evidence was judged to be low. This assessment reflects the exclusively observational study designs, small sample sizes, heterogeneity in CPET protocols and outcome reporting, and imprecision of estimates. Formal GRADE profiling was limited by the absence of pooled effect estimates, inconsistent comparator frameworks, and variable reporting of statistical measures across studies.

Additionally, although several studies reported statistically significant associations, others relied on descriptive comparisons or correlations that did not reach significance, underscoring the need for adequately powered prospective analyses. No study provided sufficiently robust data to evaluate postoperative changes in VO_2_ quantitatively.

### Risk of bias in studies

Among the studies assessed with the ROBINS-I tool**,** Shen et al. (2016) [16] and Guo et al. (2016) [18] were judged to be at serious risk of bias, while Farrell et al. (2020) [20] demonstrated a moderate risk of bias**,** and Lin et al. (2019) [17] was at low risk of bias overall. Shen et al. (2016) [16] showed uncontrolled confounding and selection bias, with unadjusted covariates such as BMI, physical activity, and smoking exposure, and exclusion of male participants reducing representativeness. Guo et al. (2016) [18] similarly exhibited residual confounding, as sex, scoliosis type, and Risser's sign were not accounted for, and its single-center, baseline-only design limited causal inference. Farrell et al. (2020) [20] demonstrated moderate risk due to partial adjustment for key variables, omitting BMI and physical activity; however, data completeness and consistent preoperative assessments supported reliability. In contrast, Lin et al. (2019) [17] applied standardized American Thoracic Society/European Respiratory Society (ATS/ERS) protocols, rigorous inclusion criteria, and validated outcome measures, with only minor residual confounding unlikely to affect results materially.

For studies appraised using the JBI Critical Appraisal Checklist**,** Siwiec et al. (2024) [8] and Lau et al. (2023) [19] were both judged to be at low risk of bias**.** Siwiec et al. (2024) [8] defined inclusion criteria clearly, applied validated radiographic and CPET methods, and controlled for confounding via age- and sex-matched controls and regression analysis. Lau et al. (2023) [19] used blinded, standardized cardiopulmonary testing and adjusted for key covariates including menarche onset, visceral adiposity, and body fat percentage. Although control participants’ spinal status was verified clinically rather than radiographically, this was applied consistently and was unlikely to influence VO_2_ outcomes.

## Discussion

This systematic review aims to establish the clinical utility of cardiopulmonary metrics as instruments to stratify and consequently monitor the recovery of patients with AIS. Although not all included studies directly measured VO_2_ max or VO_2_peak, spirometric outcomes were incorporated in accordance with the predefined protocol to provide comparative insight into static vs exercise-based cardiopulmonary assessment. Several studies have linked the anatomical deformity pathognomonic of scoliosis with impaired exercise capacity by virtue of reduced pulmonary function [1]. Aside from the mechanical disadvantage associated with reduced thoracic volume, recent studies have demonstrated cardiac structural implications of this pathology [9]. Left ventricular dimensions, cardiac index, and ejection fraction were all negatively impacted, independent of age and gender. Accordingly, CPET and metrics, such as VO2 max, serve as the instruments through which these physiological adaptations can be quantified, and more importantly for clinicians, monitored over time. Hence, this study aims to explore the current literature, synthesize recommendations in healthcare applications, and identify future directions for research.

Currently, postoperative recovery is monitored through radiological, functional, and quality-of-life outcomes. A satisfactory postoperative Cobb angle correction is typically defined as a reduction of at least 50% from the preoperative curve magnitude or achievement of a final Cobb angle less than 25° [21]. However, this value must be translated into functional improvements in the quality of life of patients for clinicians to derive an applicable notion of the impact that surgery has on the lives of patients. Additionally, the SRS-22 survey, comprising physical activity, pain, appearance, and mental satisfaction, employs patient-reported outcomes to monitor response to surgery by virtue of quality-of-life improvements [22]. It is important to emphasize that surgical management of AIS is primarily undertaken for deformity control, progression prevention, and quality-of-life improvement rather than restoration of pulmonary physiology in most cases. However, studies often overlook the value of cardiopulmonary testing in the surgical follow-up, both acutely and in the context of long-term physical rehabilitation [23].

### Cardiopulmonary adaptations

**Figure 2:**
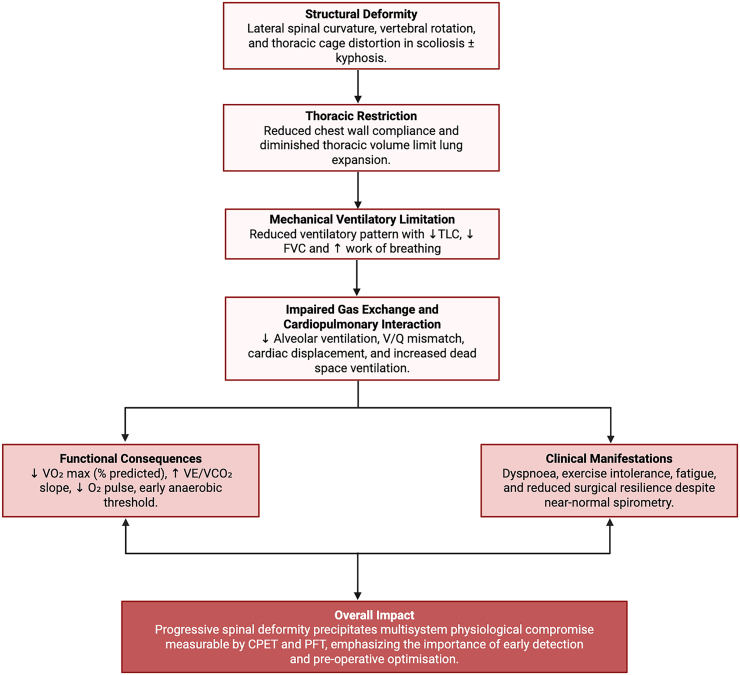
Conceptual framework depicting the cascade from structural deformity to cardiopulmonary dysfunction in scoliosis. Progressive spinal curvature and thoracic restriction precipitate ventilatory limitation, impaired gas exchange, and reduced aerobic capacity, culminating in measurable functional impairment and diminished surgical resilience. *TLC, total lung capacity; FVC, forced vital capacity; VO_2_ max, maximal oxygen uptake; VE/VCO_2_, ventilatory equivalent for carbon dioxide; O_2_, oxygen; CPET, cardiopulmonary exercise testing*.

It has been established that the altered thoracic geometry associated with scoliosis reduces chest wall compliance and limits lung expansion, culminating in restrictive ventilatory impairment (Fig. 2) [1]. Thus, our included studies showed pulmonary function tests (FEV_1_ and FVC values) that were consistently 85-90% of predicted. Aside from this association, the relationship between markers of deformity (Cobb angle, thoracic curvature) and PFTs, is especially apparent in severe thoracic curves (Cobb >80°), indicating reduced quality of life for patients with more severe irregularities. Compounding this is the even more significant reduction in VO2 max (20–30% lower) [8] compared to age-matched controls for subjects without spinal deformity. The disproportionate reduction in this integrative cardiopulmonary marker, compared to PFTs, may support the hypothesis that cardiac adaptations occur as a response to the deformity of scoliosis, independently of the respiratory consequences. Such a determination is supported by Xaio et al. (2025) [10], which employed echocardiographic analysis to demonstrate altered cardiac morphology as a direct result of spinal deformity. Nevertheless, purely quantitative indices may underestimate functional morbidity, as patients with relatively preserved lung volumes often still exhibit marked exercise intolerance [3].

From this perspective, it is unsurprising that indices of pulmonary function may only be modestly or inconsistently improved postoperatively. This underscores the gravity of these cardiopulmonary adaptations, and the lasting impact on the quality of life attainable for patients affected by AIS. Persons affected by such a disease from birth would benefit from long-term monitoring of disease activity, especially regarding these cardiorespiratory effects, which often go overlooked.

### Baseline preoperative VO_2_max

Our synthesis has established a correlation between curve severity and VO_2_max. Additionally, reduced VO_2_max values were observed despite relatively preserved oxygen saturation at rest, indicating that exercise testing reveals functional limitations not immediately apparent on static measures. This reinforces the role of VO_2_max as an integrative assessment of pulmonary mechanics, cardiovascular response, and peripheral oxygen utilization. The ability to capture this dynamic interplay between oxygen delivery and utilization affords clinicians a more holistic picture of the real-world impact that this condition has on patients, in a more quantitative context than word-of-mouth measures. The incorporation of CPET into preoperative evaluation enables stratification of surgical risk, identification of patients at heightened perioperative vulnerability, and optimization of prehabilitation strategies aimed at improving postoperative outcomes [24]. This is in accordance with the enhanced recovery after surgery (ERAS) protocol [25].

Although these findings are promising, there exists a substantial lack of research into the correlation between CPET metrics, specifically VO2max, and postsurgical recovery. Notably, there is a growing interest in evaluating longitudinal pre- and post-operative VO2 trajectories. This signifies a shift toward physiology-informed monitoring that complements radiographic correction and patient-reported outcomes.

### Postoperative changes and response to treatment

Following corrective spinal surgery, postoperative pulmonary function metrics improved only modestly and VO_2_max often remained below predicted levels [8]. This suggests that although intervention alleviates mechanical restriction, chronic alterations in chest wall compliance and deconditioning may limit full recovery of cardiopulmonary reserve. Notably, pulmonary function did not decline over a follow-up period of two years, indicating short-- to intermediate-term stability of cardiopulmonary parameters. However, in the absence of untreated longitudinal comparator cohorts, it cannot be determined whether this stability reflects a true treatment effect or an expected growth-related trajectory.

Hence, it is crucial that rehabilitation and prehabilitation strategies extend beyond structural correction to include targeted cardiopulmonary conditioning and aerobic retraining, if an enhanced quality of life is to be ensured [24]. In practical terms, prehabilitation may include moderate-intensity aerobic training (eg stationary cycling or treadmill walking 2-3 times per week), basic inspiratory muscle training using threshold devices, and supervised physiotherapy focused on thoracic mobility and posture optimization. Where formal CPET is available, exercise intensity can be individualized according to baseline VO_2_max or anaerobic threshold; in resource-limited settings, submaximal tests such as the 6MWT may guide prescription. The objective is not maximal conditioning, but measurable improvement in aerobic tolerance and respiratory efficiency prior to surgery, thereby enhancing perioperative resilience and facilitating earlier postoperative mobilization. Subsequent monitoring of such metrics throughout the postoperative course can serve as a benchmark for long-term cardiopulmonary recovery in scoliosis patients. However, this field is severely limited in terms of longitudinal prospective cohort studies evaluating such outcomes.

### Future directions

Future research should prioritize prospective, multicenter cohorts that implement standardized CPET protocols (modality, ramp cadence, termination, and effort criteria) and report VO_2_max both in mL·kg^−1^·min^−1^ and percentage predicted to establish clinically meaningful thresholds and minimal clinically important differences [26,27]. At present, no validated minimal clinically important difference for VO_2_max has been defined in pediatric scoliosis populations, limiting interpretation of whether observed preoperative differences or postoperative changes represent clinically meaningful improvement. Study designs ought to employ narrow age bands, sex-stratified analyses, and etiology-specific subgroups**,** coupled with advanced phenotyping and severity stratification of thoracic geometry (eg moderate vs severe curves, Cobb angle thresholds >80°, kyphosis, rotational indices) and cardiac evaluation (echocardiography or cardiac magnetic resonance imaging) to disentangle ventilatory from cardiovascular constraints. Prospective studies incorporating predefined perioperative endpoints–such as complication rates, length of stay, intensive care utilization, and rehabilitation milestones–are required to determine whether baseline VO_2_ max independently predicts surgical risk or recovery trajectories. At present, the use of VO_2_max for perioperative risk stratification remains investigational and requires validation through adequately powered longitudinal cohorts with multivariable modeling. Such work may also clarify whether CPET-derived metrics can provide adjunctive objective information in borderline surgical scenarios (eg skeletally mature patients with moderate curve magnitude), particularly where radiographic thresholds alone may not fully reflect functional limitation.

Randomized trials of prehabilitation targeting aerobic capacity are warranted to test whether preoperative VO_2_max augmentation reduces perioperative complications, shortens length of stay, and expedites functional recovery, with concurrent measurement of patient-reported outcomes. Given variability in CPET access, pragmatic surrogates should be validated against CPET for perioperative screening and remote longitudinal monitoring. Follow-up beyond two years is needed to delineate VO_2_ trajectories across growth and maturation and to separate surgical effects from natural history. Finally, development of a core outcome set (VO_2_max, ventilatory efficiency, complications, ERAS metrics, patient-reported outcome measures), harmonized confounder adjustment (eg BMI, habitual activity, asthma), and shared registries will enable rigorous comparative effectiveness and implementation studies, including the cost-effectiveness of CPET-guided care.

### Limitations of the study

First, there is substantial clinical and methodological heterogeneity across the included studies. The direct comparability of VO_2_max values between cohorts is limited by variations in CPET modality (cycle ergometry vs treadmill testing), exercise protocols, and the criteria used to define maximal effort. Importantly, reporting of objective maximal effort parameters (eg respiratory exchange ratio, percentage of age-predicted maximal heart rate, ventilatory reserve) was inconsistent, and in some studies VO_2_peak rather than confirmed VO_2_max was reported, limiting physiological comparability across cohorts. Similarly, inconsistencies in the reporting of variance, predicted percentages, and ventilatory parameters hinder the ability to perform quantitative synthesis, which is why a narrative approach was necessary.

Population heterogeneity further constrains interpretability. The participants in the included studies span a wide age range, with mixed scoliosis etiologies (idiopathic, congenital, and neuromuscular). Although the majority of participants had AIS, one included study examined congenital scoliosis, which may differ in thoracic development and cardiopulmonary adaptation; the limited number of studies precluded meaningful etiology-specific subgroup analysis. This introduces confounding by developmental stage, sex distribution, and baseline physical conditioning. Additionally, most included studies were conducted in China, raising the possibility of population-specific confounders such as differences in BMI distribution, environmental exposures (eg air quality or altitude), habitual physical activity levels, and genetic background, which may influence VO_2_-derived measures and limit generalizability to other populations. In addition, severity of deformity was variably reported, and few studies stratified outcomes by curve magnitude (eg >80°), kyphosis, or rotational severity; this limits interpretation of potential dose–response relationships between thoracic deformity and VO_2_-derived measures. This variability may obscure the true relationship between curve severity, thoracic restriction, and cardiopulmonary performance. Several studies also reported small sample sizes and limited longitudinal follow-up, which restricted the ability to determine the temporal trajectory of VO_2_max and pulmonary function following surgical correction. Follow-up durations rarely extended beyond two years, limiting assessment of long-term cardiopulmonary trajectory, durability of surgical benefit, or comparison with nonoperative disease progression. Moreover, detailed reporting of surgical approach (posterior, anterior, combined), extent of deformity correction (including kyphosis and rotational parameters), and degree of radiographic improvement was inconsistent or absent across studies, limiting interpretation of how specific corrective strategies may influence postoperative VO_2_ trajectories. Furthermore, due to the varying availability of CPET across institutions, the generalizability of VO_2_max-based recommendations is limited, particularly in low-resource or nonspecialist settings. Notably, the exclusion of non-English studies may have introduced language bias and limited inclusion of potentially relevant international data.

An additional limitation is that many children with scoliosis–particularly those with neuromuscular etiologies or nonambulatory status–are unable to reliably perform CPET, spirometry, or submaximal tests such as the 6MWT. As a result, the existing literature disproportionately represents ambulatory patients with idiopathic scoliosis, limiting generalizability to more functionally impaired populations. Validated alternative approaches to assess cardiopulmonary function in nonambulatory children remain scarce, highlighting a critical gap in both research and clinical practice.

Lastly, risk of bias across studies was largely attributable to residual confounding and selection bias in observational designs. While cross-sectional studies showed low risk under the JBI checklist, ROBINS-I assessments identified moderate to serious bias in cohort studies due to incomplete confounder control. Additionally, several studies relied on published normative reference values rather than contemporaneous matched control groups, which may introduce comparator bias and reduce the internal validity of effect estimates. Furthermore, most surgical cohorts inherently represent patients deemed fit for operative management, introducing selection bias and limiting extrapolation of CPET-derived metrics to higher-risk or nonoperable populations. Future research should adopt standardized CPET protocols and rigorous multivariable adjustment to enhance causal validity.

## Conclusion

This systematic review underscores the critical yet under-recognized role of cardiopulmonary assessment in scoliosis care. Adolescents with scoliosis exhibit consistent reductions in VO_2_max and exercise efficiency, reflecting integrated cardiac and pulmonary limitations that are not captured by conventional spirometry or radiological indices. The current evidence supports an association between thoracic deformity and reduced exercise capacity; however, data are insufficient to establish VO_2_max as a validated predictor of perioperative risk or postoperative recovery. Future prospective, longitudinal studies are required to determine whether CPET-derived metrics can meaningfully inform surgical risk stratification, rehabilitation planning, or long-term functional monitoring.

## Author contributions

**Suhani Sharma:** Writing – review & editing, Writing – original draft, Visualization, Validation, Project administration, Methodology, Investigation, Formal analysis, Data curation, Conceptualization. **Arun Ramnarine:** Writing – review & editing, Writing – original draft, Data curation. **Karim Fouad:** Writing – review & editing, Writing – original draft, Data curation. **Samuel Kirby:** Writing – review & editing, Writing – original draft, Data curation. **Shreya Sankar:** Visualization, Validation, Supervision, Project administration, Conceptualization. **Michael O'Connor:** Visualization, Validation, Supervision, Project administration, Conceptualization. **George-Paul O'Byrne:** Visualization, Validation, Supervision, Project administration, Conceptualization. **Oliver Boughton:** Writing – review & editing, Visualization, Validation, Supervision, Resources, Project administration, Methodology, Investigation, Funding acquisition, Conceptualization. **Patrick Kiely:** Writing – review & editing, Visualization, Validation, Supervision, Resources, Project administration, Methodology, Investigation, Funding acquisition, Conceptualization. **Tony Rafferty:** Writing – review & editing, Visualization, Validation, Supervision, Resources, Project administration, Methodology, Investigation, Conceptualization.

## Ethics approval and consent

The author(s) declare that no patient consent was necessary as no images or identifying information are included in the article.

## Funding

None to declare.

## Declaration of competing interests

The authors declare that they have no known competing financial interests or personal relationships that could have appeared to influence the work reported in this paper.

## Figures and Tables

**Table:** 

Database	Search String
PubMed	("Scoliosis/surgery"[MeSH] OR "spinal fusion"[MeSH] OR scoliosis surgery[tiab] OR spinal fusion[tiab]) AND ("oxygen consumption"[MeSH] OR VO2max[tiab] OR maximal oxygen uptake[tiab] OR peak oxygen consumption[tiab]) AND ("child"[MeSH] OR "adolescent"[MeSH] OR pediatric[tiab] OR pediatric[tiab] OR child∗[tiab] OR adolescen∗[tiab]) AND ("rehabilitation"[MeSH] OR "exercise Tolerance"[MeSH] OR "pulmonary function tests"[MeSH] OR "quality of life"[MeSH] OR "postoperative complications"[MeSH] OR functional recovery[tiab] OR postoperative recovery[tiab] OR exercise tolerance[tiab] OR QoL[tiab] OR complications[tiab])

**Table:** 

Author	Study Title	Study Design	Country	Year	Population (n, M/F)	Mean Age (Years ± SD)	BMI ± SD	Mean Pre-op VO_2_max (mL/kg/min ±SD)	VO_2_max (% Pred ± SD)	FEV_1_ (% Pred ± SD)	FVC (% Pred ± SD)	TLC (% Pred ± SD)	Thoracic Curvature (° ± SD)	Thoracic Kyphosis (° ± SD)	Results	Conclusions	Additional Notes
Shen (2016)	Cardiopulmonary exercise testing in patients with idiopathic scoliosis	Prospective cohort	China	2016	33 - F 7 - M	15.5 ± 5.0	19.20 ± 2.7	27.86 ± 5.2	79.9 ± 14	90.69 ± 16	90.03 ± 14	91.58 ± 13	49.4 ± 11	17.2 ± 12	Larger curves worsened PFTs; regular exercise improved VO_2_peak.	VO_2_peak was key for exercise capacity but unrelated to curve severity.	
Lin (2019)	Cardiopulmonary function in patients with congenital scoliosis	Observational cohort	China	2019	60 (19 M, 41 F)	18.3-20.2 ± 7.2	20.6 ± 3.14	–	70.2 ± 12.5 (A); 70.6 ± 14.7 (B); 62.0 ± 12.1 (C)	A: 83.4 ± 20.4; B: 70.5 ± 12.7; C: 53.3 ± 17.1	A: 80.6 ± 18.6; B: 70.3 ± 12.1; C: 55.8 ± 16.5	A: 79.2 ± 17.5; B: 69.2 ± 12; C: 56.3 ± 15.9	84.4 ± 38.3	52.3 ± 45.4	Deformity severity correlated inversely with VO_2_max.	Restrictive dysfunction limits exercise tolerance; VO_2_max inversely related to thoracic kyphosis.	Groups A–C based on major thoracic curve severity (40-70°, 71-100°, >100°).
Guo (2024)	Application of the six-minute walk test in assessment of cardiopulmonary function of children with idiopathic scoliosis	Cohort	China	2024	65	12.77 ± 2.07	18.30 ± 2.55	–	–	–	–	–	26.02 ± 13.76	24.04 ± 12.55	6MWT correlated positively with VO_2_max (r = 0.431, *P* < .001).	6MWT effectively assesses exercise tolerance in IS patients.	No raw CPET values reported.
Siwiec (2024)	Impact of idiopathic scoliosis on the cardiopulmonary capacity of adolescents	Cross-sectional	Finland	2024	92 (63 F, 29 M)	13.3 ± 2.46	0.7 ± 1.3	33.59 ± 7.45 (IS); 43.04 ± 8.34 (controls)	–	–	–	–	–	–	Longer test duration in IS; reduced VO_2_peak.	CPET should be used to monitor cardiopulmonary function in IS.	Compared IS vs controls.
Lau (2023)	Patients with adolescent idiopathic scoliosis have higher oxygen consumption during E-Fit	Prospective case-control	China	2023	22 F (10 AIS, 12 controls)	12.11-13.71 ± 2.0	17.9 ± 1.7	35.24 ± 5.35 (IS); 35.72 ± 3.9c (controls)	–	–	–	–	24.5 ± 6.38	–	AIS patients had higher oxygen consumption during E-Fit.	Mild–moderate AIS showed increased metabolic cost vs controls.	22 females; 10 AIS, 12 controls.
Farrell (2020)	Predicting preoperative pulmonary function in thoracic AIS from radiographic parameters	Retrospective cohort	UK	2020	170	15.1 ± 3.5	53.1 ± 13.9 (kg)	–	–	69.1 ± 15.7	72.9 ± 15.2	–	21.8 ± 16	–	Thoracic shape predicts FEV_1_/FVC better than Cobb angle.	Radiographic metrics better predictors than curvature alone.	Includes 12 adults (10-39.8 y).
